# Principles of Physiology, General and Comparative

**Published:** 1853-04

**Authors:** 


### BIBLIOGRAPHICAL.

*Principles of Physiology, General and Comparative.* By WILLIAM B. CARPENTER, M. D., F. R. S., F. G. S., Examiner in Physiology and Comparative Anatomy in the University of London, Professor of Medical Jurisprudence in University College, &c., pp. 1098. London. John Churchill, 1851.

THIS work, London edition, is illustrated with three hundred and twenty-one wood-engravings, and can be obtained of Lindsay & Blakiston, book publishers, of Philadelphia.

This great work of Dr. Carpenter is divided into two parts—the first treats of general physiology, the second of special and comparative physiology.

*General Physiology* comprises eight chapters—the

1st chapter treats of the nature and objects of the science of physiology.

2d is on the general characters of organized structures.

3d. Nature and conditions of vital phenomena.

4th. Component structures of organized fabrics.

5th. Characteristics of the vegetable and animal kingdoms.

6th. General view of the vegetable kingdom.

7th. General view of the animal kingdom.

*Special and Comparative Physiology*, comprises twenty-two chapters treating of functions, aliment, heat, light, electricity, &c.

